# A Rare Case of Tumoral Calcium Pyrophosphate Dihydrate Crystal Deposition Disease of the Wrist Joint

**DOI:** 10.1155/2015/313291

**Published:** 2015-11-17

**Authors:** Osamu Nakamura, Yoshio Kaji, Yoshiki Yamagami, Kounosuke Yamaguchi, Hideki Nishimura, Natsuko Fukuoka, Tetsuji Yamamoto

**Affiliations:** Department of Orthopaedic Surgery, Kagawa University Faculty of Medicine, 1750-1 Ikenobe, Miki-cho, Kita-gun, Kagawa 761-0793, Japan

## Abstract

*Introduction*. Tumoral calcium pyrophosphate dihydrate (CPPD) crystal deposition disease (CPPDCD), also known as tophaceous calcium pyrophosphate deposition disease (CPDD), is a tumorlike lesion, and it should be distinguished from usual CPDD that causes severe joint inflammation and arthralgia. A case of tumoral CPPDCD of the wrist joint that required differentiation from synovial osteochondromatosis is described.* Case Presentation*. The patient was a 78-year-old woman with a 5-year history of nodular lesions at the right wrist that had gradually increased in size. An excisional biopsy and a histological examination of the excised nodular lesions by hematoxylin and eosin (H&E) staining were performed, demonstrating numerous polarizable, rhabdoid, and rectangular crystals, surrounded by fibroblasts, macrophages, and foreign body-type giant cells, consistent with tumoral CPPDCD.* Conclusion*. Tumoral CPPDCD, especially at the wrist joint, is rare, and, to the best of our knowledge, only 2 articles have been published. This case seems to need further follow-up for recurrence, because tumoral CPPDCD may recur after complete or incomplete surgical excision.

## 1. Introduction

Usual CPDD is characterized by episodes of arthralgia and the presence of CPPD crystals in the synovial fluid of the affected joint [1]. Tumoral CPPDCD, also known as tophaceous CPDD, is a tumorlike lesion, and it should be distinguished from usual CPDD. It usually occurs in the temporomandibular joint, occasionally in the perispinal tissues, but rarely in the joints of the extremities [2, 3]. A rare case of tumoral CPPDCD of the wrist joint that required differentiation from a soft tissue tumor such as synovial osteochondromatosis is reported.

## 2. Case Presentation

**Figure 1:**
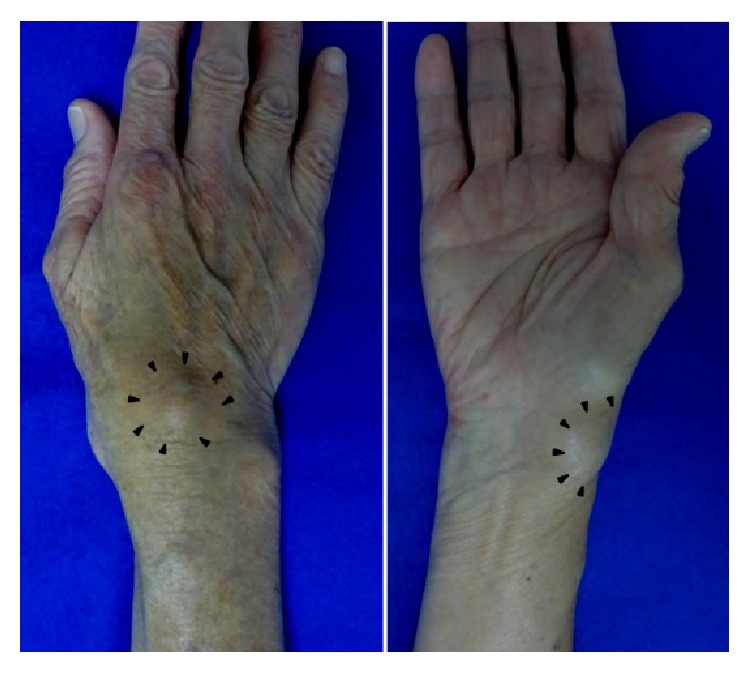
External appearance of the right wrist joint.

The patient was a 78-year-old woman with a 5-year history of nodular lesions on the right wrist. She consulted a clinic and was referred to our hospital in August 2014 with only two nodular lesions on the right wrist. On physical examination, the radial nodular lesion was 2 cm × 2 cm in size, the dorsal nodular lesion was 2 cm × 2.5 cm in size, and these nodular lesions were not movable, painful, or tender (Figure 1). She had no previous history of gout, hyperparathyroidism, hemochromatosis, or hypothyroidism. She had no episodes of trauma to her right wrist.

**Figure 2:**
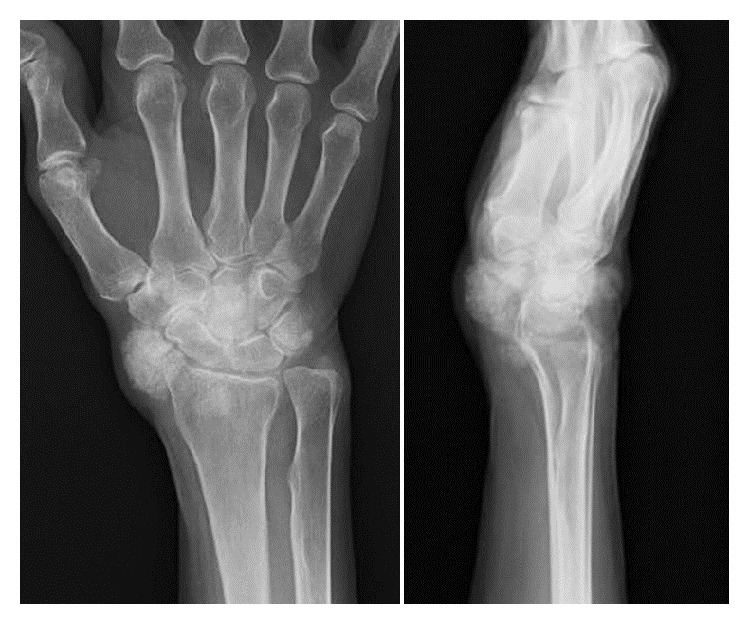
X-ray findings show some calcified nodular lesions on the right wrist joint.

**Figure 3:**
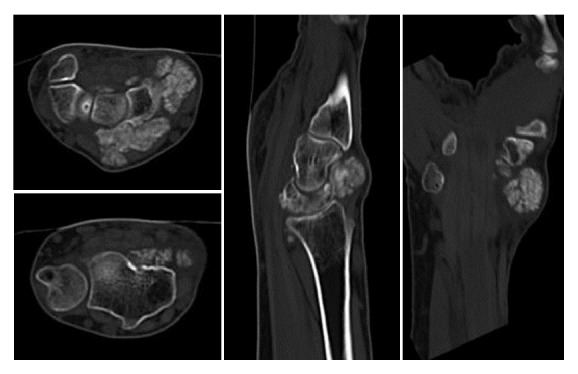
Computed tomography shows the details of the lesions.

**Figure 4:**
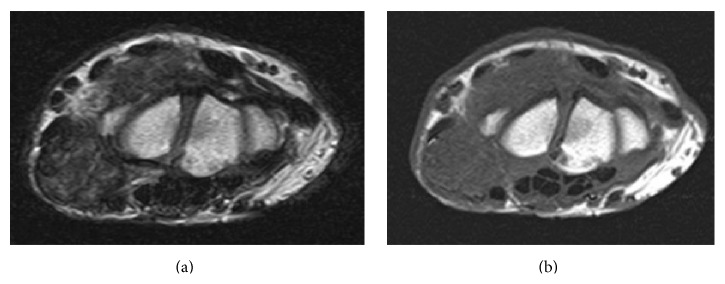
Magnetic resonance imaging reveals some nodular lesions at the wrist joint. (a) T2-weighted imaging. (b) T1-weighted imaging.

Radiographs of the right wrist joint before surgery showed multiple periarticular nodular lesions with calcifications on the volar (this lesion was not palpable on the skin), radial, and dorsal aspects of the right wrist joint (Figure 2). On computed tomography (CT), multiple calcified nodular lesions in and around the right wrist joint were shown in detail (Figure 3). Magnetic resonance imaging (MRI) revealed that these nodular lesions around the right wrist joint showed low signal intensity on T1-weighted images and a mixture of high- and isosignal intensity on T2-weighted images (Figure 4).

There were no abnormal findings on peripheral blood examination. Laboratory studies showed normal serum uric acid, calcium, phosphorus, alkaline phosphatase, and C-reactive protein levels. These clinical and radiographic findings suggested an initial diagnosis of soft tissue tumor, such as synovial osteochondromatosis, which may occur secondarily or haphazardly in combination with CPDD [2].

**Figure 5:**
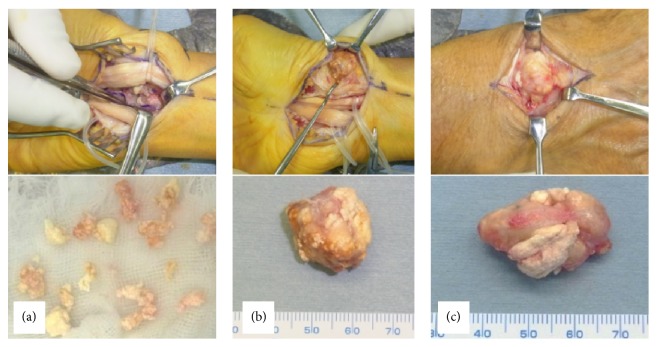
Photographs show nodular lesions of the resected specimen. (a) Volar lesions were resected piece by piece. (b) Radial lesion. (c) Dorsal lesions were resected en bloc.

An excisional biopsy was performed. The volar and radial nodular lesions were exposed via a volar incision. First, the volar lesion was excised piece by piece, avoiding the flexor tendons and the median nerve (Figure 5(a)). Next, the lesion was excised en bloc (Figure 5(b)). The lesion on the dorsal side was excised en bloc via a dorsal incision avoiding the extensor tendons and opening the 4th extensor compartment (Figure 5(c)).

**Figure 6:**
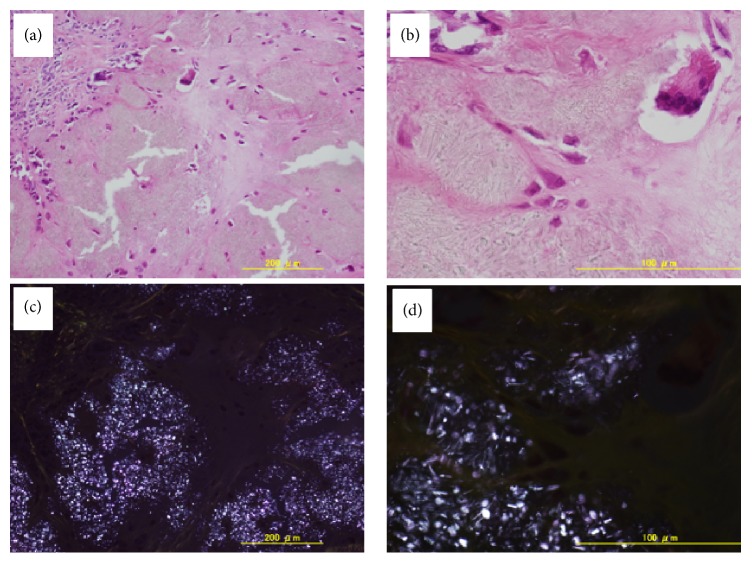
Photomicrograph of the tumoral mass shows deposits of crystals surrounded by fibroblasts, macrophages, and foreign body-type giant cells (H&E, (a) low-power view, (b) high-power view). Under polarized light, the calcified deposits show weekly positive birefringence suggestive of CPPD ((c) low-power view, (d) high-power view).

On histological examination of the excised tumor tissue with H&E staining, numerous polarizable, rhabdoid, and rectangular crystals, surrounded by fibroblasts, macrophages, and foreign body-type giant cells, were seen (Figures 6(a) and 6(b)). The calcified deposits showed weakly positive birefringent polarized light, suggestive of CPPD crystals, and these findings were consistent with tumoral CPPDCD (Figures 6(c) and 6(d)). The slides were examined using a Zeiss LSM 710 microscope (Carl Zeiss, Munich, Germany).

**Figure 7:**
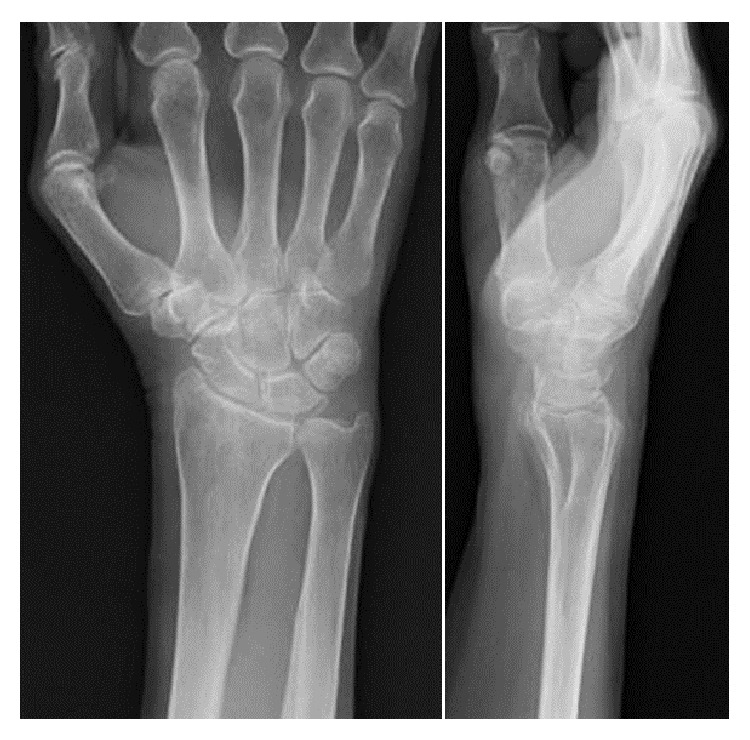
X-ray findings at last follow-up, 6 months after surgery.

At 6-month follow-up, she had no swelling of her right wrist, and the radiographs showed no evidence of recurrence (Figure 7).

## 3. Discussion

CPPD crystals were first identified in 1961 in the synovial fluid of patients with gout-like symptoms without sodium urate crystals, which were described by McCarty as CPDD [4]. The risk factors for CPDD are aging, previous trauma to the affected joint including surgery, and certain metabolic diseases, such as hyperparathyroidism, hypothyroidism, and hemochromatosis. Tumoral CPPDCD is one of the rarest forms of CPDD, characterized by focal deposition of CPPD and formation of a mass [5]. CPDD usually affects larger joints such as the knee, shoulder, wrist, or ankle. On the other hand, Yamakawa et al. reported that the most common anatomic location of tumoral CPPDCD is the temporomandibular joint, followed by the cervical spine and hand. Moreover, based on a review of the reported cases (54 cases), which included their series, they proposed that the lesions of tumoral CPPDCD could be divided into two categories: the central (head and neck) type (33 cases) and the distal (extremity) type (21 cases). In these review cases, only 2 cases occurring on the wrist joint were reported. They also showed that these two groups of tumoral CPPDCD were not different with respect to age and sex distributions but showed different clinical symptoms. In the central type, the most common symptom was the presence of a painful mass. In the distal type, the most common symptom was a painless mass or swelling [3]. In this case study also, the patient had a painless mass. Furthermore, because there was no increase in inflammatory findings as in CPDD, it was difficult to differentiate the present lesions from tumor in this case. In addition, tumoral CPPDCD, especially at the wrist joint, is rare, with, to the best of our knowledge, only 2 articles having been published: Ohshio et al. in 1991 [6] and Yamakawa et al. in 2001 [3].

Tumoral CPPDCD should be differentiated from tumoral calcinosis and malignant or benign tumors such as synovial chondromatosis. The location of tumoral calcinosis is similar to that of tumoral CPPDCD but it occurs predominantly in adolescents and young adults and is more often multiple than solitary. On the other hand, tumoral CPPDCD is usually seen in elderly patients. Histopathologically, the calcified material in tumoral calcinosis lacks a crystalline structure and is composed mainly of hydroxyapatite [7], which differs from tumoral CPPDCD. Unlike tumoral calcinosis, tumoral CPPDCD has a crystalline appearance when examined by polarizing microscopy [8]. Synovial chondromatosis can be confused with tumoral CPPDCD, especially when heavily calcified cartilage matrix may obscure its cartilaginous nature. Synovial osteochondromatosis is characterized by the formation of various sizes of cartilaginous or osteocartilaginous nodules in the synovial membrane and the frequent occurrence of free-floating bodies within the joint space [9].

Tumoral CPPDCD may recur after complete or incomplete surgical excision. Ishida et al. reported that one of five cases of tumoral CPPDCD had recurrences on two occasions during a 20-year span [7]. Therefore, this case seems to need further follow-up for recurrence.

## 4. Conclusion

In summary, this report described a rare case of tumoral CPPDCD at the wrist joint. Preoperative clinical and radiographic findings suggested an initial diagnosis of synovial osteochondromatosis, and an excisional biopsy was performed. However, the histological examinations were consistent with tumoral CPPDCD. Because tumoral CPPDCD is a rare disease and it is difficult to diagnose preoperatively, an excisional biopsy is necessary for pathological diagnosis.
